# The Effect of Fatty Acids on Ciprofloxacin Cytotoxic Activity in Prostate Cancer Cell Lines—Does Lipid Component Enhance Anticancer Ciprofloxacin Potential?

**DOI:** 10.3390/cancers14020409

**Published:** 2022-01-14

**Authors:** Alicja Chrzanowska, Wioletta Olejarz, Grażyna Kubiak-Tomaszewska, Andrzej K. Ciechanowicz, Marta Struga

**Affiliations:** 1 Chair and Department of Biochemistry, Medical University of Warsaw, ul. Banacha 1, 02-097 Warsaw, Poland; mstruga@wum.edu.pl; 2 Department of Biochemistry and Pharmacogenomics, Faculty of Pharmacy, Medical University of Warsaw, 02-097 Warsaw, Poland; wolejarz@wum.edu.pl (W.O.); grazyna.kubiak-tomaszewska@wum.edu.pl (G.K.-T.); 3 Laboratory of Regenerative Medicine, Center for Preclinical Research and Technology, Medical University of Warsaw, 02-097 Warsaw, Poland; andrzej.ciechanowicz@wum.edu.pl

**Keywords:** ciprofloxacin conjugates, fatty acids, prostate cancer, cytotoxic activity, proteomic analyses

## Abstract

**Simple Summary:**

Most prostate cancers are initially hormone-dependent but later gain a hormone-independent phenotype associated with changes in lipid metabolism, including enhanced absorption of extracellular fatty acids. The aim of our study was to assess the effect of ciprofloxacin conjugates with fatty acids on different type of prostate cancer (LNCaP and DU-145) and normal (RWPE-1) cells, as well as their influence on cell lipid metabolism by proteomic analysis. All tested conjugates exhibited cytotoxic potential, the most powerful for oleic, elaidic and docosahexaenoic acids. The hormone-independent DU145 line was more sensitive to derivatives than the hormone-dependent LNCaP line. These results are consistent with previously observed pronounced cytotoxic effect of conjugates on a hormone-insensitive PC3 line. Tested derivatives decreased intensity of proteins involved in prostate cancer lipid metabolism. Our findings confirm the involvement of lipid metabolism in prostate carcinogenesis indicating a target for fatty acids as drug carriers.

**Abstract:**

Purpose: To assess cytotoxic effect of ciprofloxacin conjugates with fatty acids on prostate cancer cells (LNCaP and DU-145) with different hormone sensitivity, based on previous promising results from the PC3 cells. Methods: Cytotoxicity were estimated using MTT and LDH tests, whereas its mechanisms were estimated by apoptosis and IL-6 assays. The intensity of proteins involved in lipid metabolism was determined using ML-CS assay. Results: The hormone insensitive DU-145 cells were more vulnerable than the hormone sensitive LNCaP cells. The IC50 values for oleic (4), elaidic (5) and docosahexaenoic acid (8) conjugates were 20.2 µM, 17.8 µM and 16.5 µM, respectively, in DU-145 cells, whereas in LNCaP cells IC50 exceeded 20 µM. The strong conjugate cytotoxicity was confirmed in the LDH test, the highest (70.8%) for compound (5) and 64.2% for compound (8) in DU-145 cells. This effect was weaker for LNCaP cells (around 60%). The cytotoxic effect of unconjugated ciprofloxacin and fatty acids was weaker. The early apoptosis was predominant in LNCaP while in DU-145 cells both early and late apoptosis was induced. The tested conjugates decreased IL-6 release in both cancer cell lines by almost 50%. Proteomic analysis indicated influence of the ciprofloxacin conjugates on lipid metabolic proteins in prostatic cancer. Conclusion: Our findings suggested the cytotoxic potential of ciprofloxacin conjugates with reduction in proteins involved in prostate cancer progress.

## 1. Introduction

The prostate gland is an organ that is frequently affected by disease in the male population. While in young men the dominant disease of the prostate is prostatitis, older people (over 40 years of age) are more likely to develop benign prostate hyperplasia (BPH) and glandular cancer-prostate cancer (PCa) [1]. Along with lung and colorectal cancers (CRCs), prostate cancer is the most commonly diagnosed cancer, accounting for more than 1 in 5 new diagnoses [2]. Most prostate cancers are androgen dependent at the beginning of their development, which allows for an efficient and effective androgen deprivation therapy. However, over time, some cancer cells are able to survive and grow during this therapy, resulting in an androgen-independent type because of the reactivation and abnormal activation of the androgen receptor (AR). At this point, the disease called castration-resistant PCa (CRPC) is fatal as there are no effective targeted therapies available [3].

Oncological patients undergoing chemotherapy or radiotherapy may receive ciprofloxacin as a part of the treatment for numerous infections. This quinolone is used because of the effectiveness of the therapy and its safety. Ciprofloxacin can be used to prevent bacterial infections in patients with prolonged neutropenia or acute leukemia and also after bone marrow transplantation. Despite the fact that the main therapeutic application of ciprofloxacin is the therapy of bacterial infections, many studies are conducted to determine its antiproliferative and antiapoptotic properties on selected cancer cell lines [4].

It was researched that, besides bacterial enzymes, this chemotherapeutic agent also inhibits eukaryotic topoisomerase. It can cause apoptosis of neoplastic cells by arresting the cell cycle and by inducing breaks in the DNA double-strand structure [5].

Ciprofloxacin (CP) has the cytotoxic potential to affect prostate cancer cells as shown in in vitro studies. The use of this quinolone in a therapeutic dose inhibits the growth of cancer cells and at the same time does not affect the normal cells of the prostate epithelium. Compared to other drugs used in modern chemotherapy, ciprofloxacin exhibits low toxicity [6]. Doxorubicin and docetaxel are drugs commonly used in the treatment of hormone sensitive prostate cancer. In order to determine the effectiveness of their action on cancer cell lines, these drugs, along with ciprofloxacin, have been tested in in vitro studies, both in various configurations as well as independently. It has been shown that ciprofloxacin sensitizes neoplastic cells to doxorubicin and docetaxel [7].

Modification of the lipophilicity of drugs can improve the pharmacokinetic profile. In addition, most of the drugs used in cancer treatment have a hydrophilic nature and enter into the cancer cells by active transport. The mechanisms of resistance of the cancer cells to this type of active substance develop very quickly, which is caused by dysregulation of the receptor activity in cell membranes. In order to improve the transport of the active substance to the site of action, a number of modifications are used. The chemical bond between the drug and the fatty acid allows the drug substance to penetrate the tumor cell to a greater extent. The drugs produced in this way are transported inside the tumor cell without the use of a carrier, bypassing the resistance mechanisms of these cells. The anti-tumor properties of the drug are also reduced due to the efflux phenomenon. Removal of drugs from cancer cells is accomplished by a number of transporters, including P-glycoprotein or multi-drug resistance protein (MDR). Medicinal substances whose lipophilicity has been increased are less affected by cell efflux [8,9].

Modifications of commonly used anticancer drugs by coupling them with fatty acids (FAs) of different chain length and saturation degree may improve their tissue selectivity and potentially affect the chemotherapy efficacy and safety. Combinations with fatty acids improve drug stability and hence bioavailability, limiting undesirable interactions and increasing the circulatory half-life. Because FA conjugates are not cytotoxic for normal cells, they can minimize side effects and improve the quality of chemotherapy [10]. Cytosine arabinoside is characterized by rapid elimination of the active triphosphate form and therefore binding of this drug to long-chain (C16–C20) fatty acids increased its antitumor activity. The modified drug is transported inside the cell by a mechanism independent of the nucleoside transporter and is degraded to a lesser extent by deaminase. It was also found that the hydrolysis rate of cytosine arabinoside conjugates with the above-mentioned fatty acids is inversely proportional to the fatty acid chain length [11].

Other conjugated nucleosides such as floxuridine and gemcitabine were more intensely transported through the membranes. In order to improve the therapeutic properties of the conjugation with lipid molecules, also cytostatics from the group of antibiotics, such as mitomycin C or doxorubicin are applied [12]. In vitro and in vivo studies have confirmed the effectiveness of fatty acids in the treatment and prevention of breast cancer. Stearic acid inhibits the proliferation of breast cancer cells and induces apoptosis. In order to achieve greater effectiveness of the therapy, stearic acid was combined with the propofol molecule. This combination had a strong growth inhibitory effect and inhibited the migration and adhesion of neoplastic cells, which is not observed when these compounds were used alone [13].

In vitro studies on cell lines derived from prostate cancer have been conducted for decades and are a source of knowledge about the prostate cancer pathogenesis. The three following epithelial cell lines, isolated from the most common sites of metastasis, are often used as a useful model for prostate cancer examination. The PC3 line first described by Kaighn is derived from bone metastases and is characterized by high invasiveness (stage IV) [14]. The DU145 line is derived from brain metastases isolated by Stone [15]. The cells of this line are characterized by a relatively low invasiveness compared with PC3 cells (stage II). Both the PC-3 and DU-145-lines are insensitive to hormone therapy. LNCaP is characterized by a relatively slow growth of cells isolated for the first time by Horoszewicz from metastases of prostate cancer to supraclavicular lymph node [16]. This line differs from PC3 and DU-145 because of the lower invasiveness degree and sensitivity to hormonal treatment. The RWPE-1 cell line was isolated from prostate epithelial cells with normal histology. These cells were transfected with a human papillomavirus 18 single copy for the immortalization. The androgen receptor is present on RWPE-1 cells and its expression increases upon exposure to androgen hormones [17].

Prostate cancer (PCa) is well known as a lipid-dependent tumor [18]. It is interesting that prostate cancer cells, unlike other types of cancer, mainly use fatty acids as an energy substrate and are highly effective in obtaining fatty acids from the external environment. The lipid profile in prostate cancer cells provides cells with saturated and mono-unsaturated acyl chains, which replenishes the cells with membrane components. During certain metabolic stress, prostate cancer cells may switch metabolic pattern from de novo fatty acids (FA) synthesis to scavenging extracellular lipids [19,20,21]. PCa, as one of several cancers, is characterized by abnormal lipid accumulation in the form of lipid droplets (LDs). This process could be closely related to the progression of CRPC and drug resistance through increasing intra-tumoral androgen synthesis, which promotes androgen receptor (AR) reactivation and abnormal activation [22,23]. Taken together, prostate cancer exhibits metabolic plasticity in acquiring lipids by uptake, indicating potential therapeutic benefit by co-targeting lipid supply or using lipids as carrier for anticancer agents.

In our previous studies we evaluated cytotoxicity, apoptosis-inducing effects and interleukin 6 (IL-6) inhibition release in human primary and metastatic colon cancer (SW480, SW620), metastatic prostate cancer (PC3) and normal keratinocytes (HaCaT) cell lines after treatment with ciprofloxacin conjugates [24]. It was established that prostate cancer cells are more sensitive for fatty acids—ciprofloxacin amides. Now, we studied the effect of ciprofloxacin conjugates on different type of prostate cancer cells (LNCaP and DU-145) as well as on the normal prostate epithelial cell line (RWPE-1). Additionally, we made an initial attempt to test their influence on lipid metabolism in cancer cells by determination of selected proteins intensity.

## 2. Materials and Methods

### 2.1. Materials

#### 2.1.1. Ciprofloxacin Derivatives

**Figure 1:**
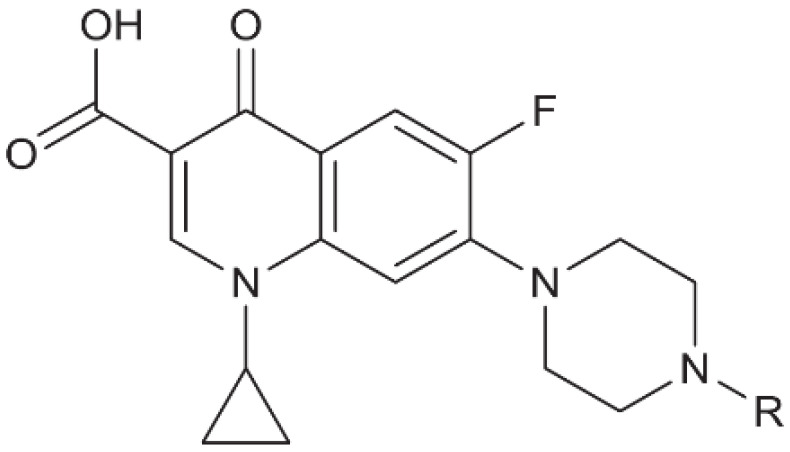
Structure of the tested compounds. (R is the fatty acid bounded by amide bond to the ciprofloxacin molecule.)

Nine newly synthesized fatty acid derivatives of ciprofloxacin were assessed for their potential antitumor activity. The ciprofloxacin molecule was linked by an amide bond with saturated and unsaturated fatty acids. The synthesis of the tested compounds takes place using the nitrogen atom from the piperazine group bound to the quinolone skeleton at position 7 of ciprofloxacin and the carboxyl group of the fatty acid (Figure 1). A detailed synthesis procedure with confirmation of the obtained products is described in our previous research [24].

The fatty acids used as starting materials in the above synthesis can be classified into three groups due to their chain length. Among the tested acids there are short-chain acids, i.e., crotonic and sorbic acid, medium-chain acid, i.e., geranic acid, while the representatives of long-chain acids are oleic, elaidic, linoleic, erucic, docosahexaenoic and palmitic acids. The fatty acids used are characterized by the presence of at least one unsaturated bond in the aliphatic chain, excluding palmitic acid that includes only saturated bonds. The used acids can also be divided according to the type of geometric isomerism. Linoleic, oleic, erucic, docosahexaenoic acids are Z isomers, while crotonic, sorbic, geranium and elaidic acids are E isomers (Table 1).

#### 2.1.2. Cell Culture

The studies were performed on the metastatic prostate cancer cell lines (LNCaP) and (DU-145) as well as on the normal prostate epithelial cell line (RWPE-1). All studied cell lines were purchased from the American Type Culture Collection (ATCC, Rockville, MD, USA). Dulbecco’s Modified Eagle’s Medium (DMEM, Biowest SAS, Nuaillé, France) supplemented with 1% streptomycin and penicillin and 10% heat-inactivated fetal bovine serum (FBS, Gibco BRL San Francisco, CA, USA) was used. The cell cultures were conducted under strictly defined conditions according to recommended ATCC protocol. Cells were incubated until the appropriate confluence was achieved (80–90%). Next, they were harvested by treatment with 0.25% trypsin (Gibco BRL, San Francisco, CA, USA) and used for studies. Cell culture plates consisted of 6, 12 or 96 wells. All experiments were repeated three times. The untreated cells were used as a control.

### 2.2. Methods

#### 2.2.1. MTT Assay

All ciprofloxacin fatty acid derivatives were tested at various concentrations (ranged from 5 to 140 µM) and leading cytostatics doxorubicin and cisplatin were added on 96-well plates (1 × 10^4^ cells per well) with seeded LNCaP, DU-145 or RWPE-1 cells and incubated for 72 h. MTT analysis was performed as described previously [24,25].

Cell absorbance results were substituted into the formula for the relative MTT level (%). It allows to calculate the viability of cells exposed to the test compounds. Cell viability is the percentage of MTT reduction in cells to which the test compounds were added compared to the control sample, where only the medium was added to the cells.

The relative MTT level was calculated using the formula: life time [100%] = A/B × 100% where: A—the test sample absorbance; B—the control sample absorbance.

#### 2.2.2. LDH Test

Selected conjugates and ciprofloxacin (CP) alone were examined at four concentrations of 10, 20, 40 and 60 µM. Lactate dehydrogenase (LDH) as a cytosolic enzyme determined in culture medium is a marker of cell death. The LDH activity determination was performed after 72 h incubation of cells (1 × 10^4^ cells per well) in 96-well plates with the selected conjugates of gradually decreasing concentration according to the procedure mentioned at earlier study by Chrzanowska et al. [24,26]. The cytotoxicity of test compounds was expressed as % of LDH released from test cells. This parameter is calculated using the formula: cytotoxicity = [A test sample − A “low control”]/[A “high control” − A “low control”] where: A—absorbance, “Low control”—cells in DMEM medium with 2% FBS, “High control”—cells in DMEM with 2% FBS with 1% Triton X-100.

#### 2.2.3. Apoptosis Detection

To determine the number of cells in early apoptosis, late apoptosis or necrosis, LNCaP, DU-145 and RWPE-1 (2 × 10^5^ cells per well) cell lines were cultured with three selected compounds **4**, **5** and **8** with IC50 concentration in 6-well plates and incubated for 72 h. Then, a commercially available kit (FITC:Annexin V Apoptosis Detection Kit I; BD Biosciences Pharmingen. San Jose, CA, USA) was used. The medium was removed from the plates and approximately 1 mL of PBS was added to the wells for rinsing, followed by 0.7 mL of trypsin, and left for 3 min. Then, the plate wells were washed twice with 0.5 mL PBS with 2% FBS and next the entire content of the given walls was transferred to test tubes. The tubes were centrifuged for 7 min in a centrifuge at 1700 rpm. The supernatant solution was removed. An amount of 2 μL of annexin and 7-AAD (7-Aminoactinomycin D) were added to the test tubes, the mixture was vortexed and then left in the dark for 15 min. After this time, 0.4 mL of PBS with FBS was added to the tubes and mixed again. The effect of the LNCaP, DU-145 and RWPE-1 cells treatment with tested compounds was analyzed by flow cytometry (Becton Dickinson). Live cells with an undamaged membrane did not show affinity for the dye, while dead and damaged cell membranes were permeable to it. Annexin V-reactive cells, but not 7-AAD-reactive cells were in the early stage of apoptosis, while cells reactive with both reagents were in late-stage apoptosis or were dead [27].

#### 2.2.4. Interleukin-6 Assay

The sandwich ELISA assay is used to confirm the presence of interleukin 6 (IL-6) in the test cell line supernatant [28]. Interleukin IL-6 ELISA kit was purchased from Diaclone SAS (Besancon Cedex, France). LNCaP, DU-145 and RWPE-1 (1 × 10^5^ cells per well) were treated with IC50 concentration of selected ciprofloxacin conjugates **4**, **5** and **8** in 12-well plates for 72 h. Before starting the test, a standard curve was prepared. An amount of 100 μL of test supernatant samples from the RWPE-1, LNCaP and DU145 cell lines incubated with the tested compounds, control solutions and blank were added to the plate’s wells. Then, 50 µL of diluted biotinylated anti-human interleukin-6 antibody was added to each well. The prepared plate was incubated at room temperature for one hour. At the end of this time, the solution in the plate wells was decanted and rinsed with the washing solution. After repeating the procedure three times, 100 μL solution of streptavidin covalently bound to horseradish peroxidase (HRP) was added to the wells. Then, the plates were incubated for 30 min. The next step was to remove the solution and rinse the plates three times. After adding 100 μL of tetramethylbenzidine (TMB) solution to each well, the plates were incubated for 15 min in the dark. In order to stop the reaction, 100 μL of H_2_SO_4_ solution were added. The absorbance of the tested samples was read using a spectrophotometer at 450 nm (Microplate Spectrophotometer Thermo Scientific™ Multiskan™ GO).

#### 2.2.5. Proteomic Analysis

Enzymes involved in lipid metabolism were analyzed in the cell lysates obtained after treatment of the cells with selected conjugates for 24 h. Cells were washed with PBS and harvested, then centrifuged at 1000× *g* for 10 min. Then, lysis buffer (containing protease inhibitor, 1% RIPA Lysis and Extraction Buffer (ThermoFisher, Waltham, MA, USA) and cold PBS were added and samples were sonicated three times in an ice bath. Next, the cell lysates were centrifuged at 14,000× *g* at 4 °C for 15 min and then supernatants were stored at 70 °C before use. Protein concentration was measured by the Bradford method.

Normalized 5 µg of proteins from each cell lysate was precipitated by ice cold (−20 °C) Acetonitrile (ACN, Merck, Kenilworth, NJ, USA, in ratio 1:4) in order to purify the proteins. To remove lysis buffer ingredients, samples were centrifuged (−9 °C, 30 min, 18,000× *g*), the supernatant was discarded and ACN excess was evaporated using a concentrator (5 min, room temp.) (Eppendorf). The dried protein pellet was dissolved in 40 mM ammonium bicarbonate. In order to increase the level of protein denaturation, 500 mM dithiothreitol (DTT, with final concentration of 20 mM) and 1 M iodoacetamide (IAA, with final concentration of 40 mM) was used for reduction and alkylation processes. After 16 h incubation in 37 °C with Trypsin Gold (Promega), digested protein samples were diluted with 0.1% formic acid (ThermoFisher) and centrifuged (+2 °C, 30 min, 18,000× *g*).

LC-MS analysis was carried out with the use of the nanoUHPLC (nanoElute, Bruker, Billerica, MA, USA) coupled by CaptiveSpray (Bruker) to an ESI-Q-TOF mass spectrometer (Compact, Bruker). Two-Column separation method was used, i.e., guard column (300 µm × 5 mm, C18 PepMap 100, 5 µm, 100 Å, Thermo Scientific, Waltham, MA, USA) and Aurora separation column (75 µm × 250 mm, C18 1.6 µm) (IonOpticks, Fitzroy, Australia) in a gradient 2% B to 35% B in 90 min with the 18 µL/h flow rate. Mobile phases: A—0.1% formic acid in water and B—0.1% formic acid in ACN were used.

Samples ionization were performed at a temperature of 150 °C, a gas flow of 3.0 L/min, and the capillary voltage of 1600 V. The ions were analyzed in the positive polarity mode in the range 150–2200 *m*/*z*, with the acquisition frequency of the 1 Hz spectrum, as well as with the autoMS/MS system. All spectra were calibrated to lock mass calibrant (1222 *m*/*z*) (Agilent, Santa Clara, CA, USA) and NaFA cluster calibrant.

**Scheme 1:**
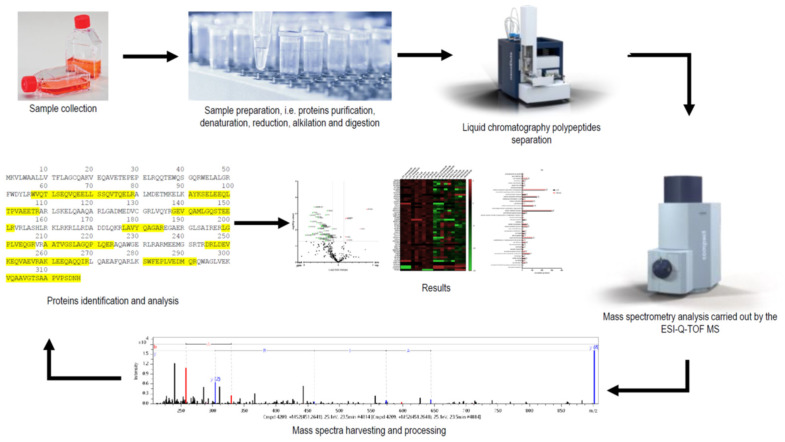
Steps of proteomic analysis.

The collected spectra were analyzed and calibrated using DataAnalysis software (Bruker) and then, identified in ProteinScape (Bruker) by the MASCOT server. Protein identification was conducted using the online SwissProt database, and their references and biological significance were identified using KEGG, String.org and Reactome.org. (Scheme 1).

#### 2.2.6. Statistical Analysis

The statistical analysis was performed using the Statistica 13.0 (StatSoft, Inc, Tulsa, OK, USA) program. Comparison between studied groups was performed by the paired Student’s t-test. Results were expressed as means ± SD, and considered statistically significant at *p* < 0.05. All data were calculated from 3 separate experiments. IC50 values were calculated using GraphPad Prism 6 software (GraphPad Software, San Diego, CA, USA).

## 3. Results

### 3.1. Cytotoxic Activity

#### 3.1.1. MTT Assay

- Effect of conjugated ciprofloxacin with fatty acids

The IC50 results from the PC3 line has been taken from our previous study and with those currently obtained are summarized in Table 2 [24]. Comparing the obtained IC50 values of the tested cell lines, it was noticed that these values are higher for the normal cell as compared to the cancer cell lines. The anticancer potential of all tested conjugates was significantly higher than the parent CP where IC50 for both tested cell lines exceeded 70 µM and for PC3 cells even 100 µM. Considering both currently tested tumor cell lines, it was observed that the IC50 values for DU145 cells are lower than those for LNCaP cells. The highest cytotoxic effect on the LNCaP cell line showed Z-polyunsaturated long-chain fatty acids DHA amide (**8**) (21.4 ± 2.2 µM) as well as conjugations with both Z/E isomers of the monounsaturated, as follows: oleic (**4**) (24.7 ± 4.1 ± µM) and elaidic (**5**) (22.7 ± 3.1 µM) acids. The lowest cytotoxicity for all studied cell lines exhibited the conjugate with shorter-chain unsaturated acid (crotonic-**1**). Similarly, acids with a short- and medium-chain and additional double bond–sorbic (**2**) and geranic (**3**) also were characterized by a much lower cytotoxicity for the LNCaP line. Among the long-chain acids, erucic (**7**) with one double bound similarly to the previously mentioned acids showed a poor cytotoxic potential. The saturated palmitic acid (**9**) and unsaturated linolenic acid derivatives expressed moderate cytotoxicity. Similarly for the DU-145 cells, the combination of ciprofloxacin with oleic, elaidic and DHA acids gave the lowest IC50 concentrations (20.2 ± 1.9, 17.8 ± 1.6 and 16.5 ± 1.4 µM, respectively). The cytotoxic effect of the remaining acids was unsatisfactory and remained at an average level. The weakest effect again has been given by the geranic and palmitic acid The selectivity index values for the DU145 cell line are higher than those for the LNCaP cell line. For the DU145 cell, the SI values for the most conjugates were above 2, whereas for LNCaP cells it was only for the conjugates **4**, **5** and **8**. Based on the above results, three compounds (marked with numbers **4**, **5** and **8**) showed the highest cytotoxicity and thus they were selected for further studies.

- Effect of unconjugated ciprofloxacin and fatty acids

To check whether the conjugation process was justified, the effect of the component conjugates added separately, i.e., being together in the mixture added to the cells culture, was also tested. Conjugate ingredients were added at corresponding concentrations that were used for conjugated compounds. In almost all cases the cytotoxic effect was weaker than for the conjugated form. Only two acids, geranic and palmitic showed a slightly higher cytotoxicity acting alone with ciprofloxacin. Interestingly, it was established that the IC50 values obtained for the free ciprofloxacin are higher than for its mixture with an addition of separate fatty acid (Table 3).

#### 3.1.2. LDH Assay

- Effect of conjugated ciprofloxacin with fatty acids

**Figure 2:**
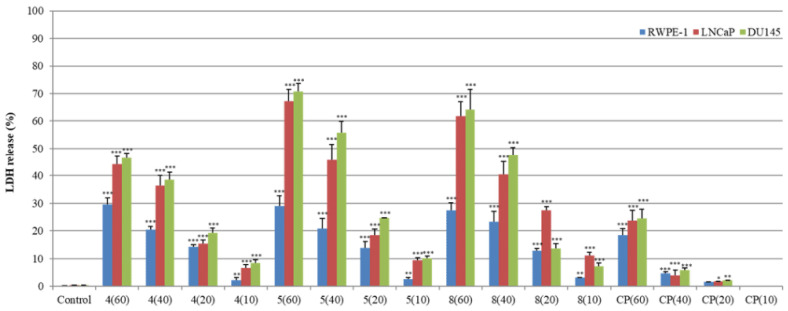
The amount of released lactate dehydrogenase (%) in cancer (LNCaP and DU145) and normal (RWPE-1) cell lines. Conjugates with oleic (**4**), elaidic (**5**) and DHA acid (**8**) and free ciprofloxacin (CP) were tested at concentrations of 60 µM, 40 µM, 20 µM and 10 µM. Significance levels explanation *** *p* < 0.001; ** *p* < 0.01;* *p* < 0.1 as compared to the control.

The values of the results obtained after conducting the LDH test enable the determination of cytotoxicity degree of the tested compounds by measuring the lactate dehydrogenase amount released from dead cells (Figure 2). This study was conducted for compounds **4**, **5** and **8** with the highest cytotoxic potential, and therefore the lowest IC50 as assessed by the MTT test.

The elaidic acid conjugate (**5**) was characterized by the highest percentages of LDH release, while the lowest value of this parameter was found for the oleic acid derivative for both tumor cell lines (DU145 and LNCaP) compared to the normal RWPE-1 line. Additionally, all tested compounds (**4**, **5** and **8**) expressed higher LDH release compared to the results obtained for ciprofloxacin alone.

Cytotoxicity, expressed as percentage of released lactate dehydrogenase, for DU145 cells was 46.5% for compound (**4**), 70.8% for compound (**5**) and 64.2% for compound (**8**). For the LNCaP cells the cytotoxicity of compounds **4, 5** and **8** were 44.2%, 67.3% and 61.8%, while for RWPE-1 cells accounted for 29.6%, 29.8% and 37.5%, respectively. The free ciprofloxacin effect was 2–3 times lower than for the conjugated form.

- Effect of unconjugated ciprofloxacin and fatty acids

**Figure 3:**
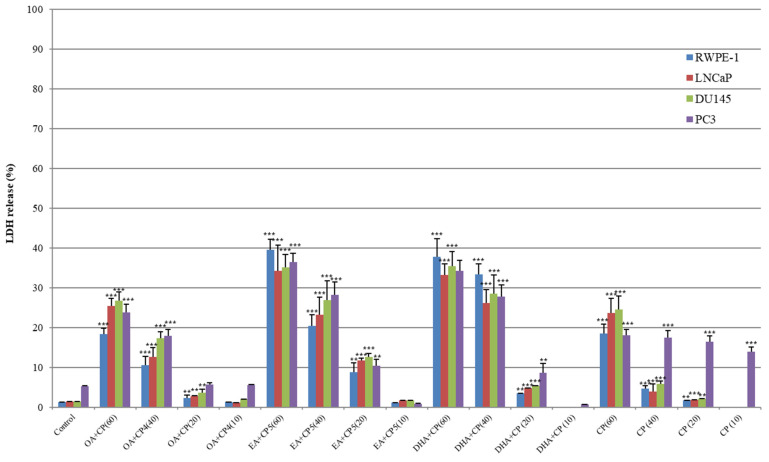
The amount of released lactate dehydrogenase (%) depending on the concentration of a mixture of ciprofloxacin (CP) and individual fatty acid OE—oleic acid; EA—elaidic acid and DHA -docosahexaenoic acid in cancerous LNCaP, DU145 PC3 and normal RWPE-1 cell lines. Tested compounds were tested at concentrations of 60 µM, 40 µM, 20 µM and 10 µM. Significance levels explanation *** *p* < 0.001; ** *p* < 0.01.

The weaker cytotoxic effect demonstrated by the MTT test for ciprofloxacin and the fatty acid mixture was also confirmed by the LDH test. Release of cytosolic enzyme LDH was quite high for DU145 cells where it accounted for 26.8%, 35.2% and 35.5% for the mixture oleic, elaidic and DHA acids together with free ciprofloxacin, respectively. For LNCaP cells the release was similar and accounted for 25.4%, 34.3% and 33.2%. Interestingly for RWPE-1 cells this release after treatment with the mixture of elaidic or DHA acid and CP was even higher than for cancer cells and accounted for 39.5% and 37.8%, respectively (Figure 3). The LDH assay again showed a weaker effect of ciprofloxacin alone on LNCaP and DU-145 cells than with the addition of selected fatty acids (range from 18.5–24.6%). The release of lactate dehydrogenase from PC3 cells under the individual fatty acid and ciprofloxacin mixture action had a slightly different pattern. Here, as in other studied cell lines, the greatest release of dehydrogenase was in the presence of elaidic and DHA acid and CP combination (36.4% and 34.2%, respectively) but a higher LDH release was observed under free CP action at concentration 20 and 10 µM as compared to mixture with fatty acid.

### 3.2. Mechanism of Conjugates Cytotoxicity

#### 3.2.1. Apoptosis

The apoptosis estimation is aimed at determining the percentage of cells that underwent the process of apoptosis after incubation with the tested compounds using flow cytometry. The results of this study make it possible to distinguish cells that are in the early phase of apoptosis from cells that are in the late phase of apoptosis or necrosis.

**Figure 4:**
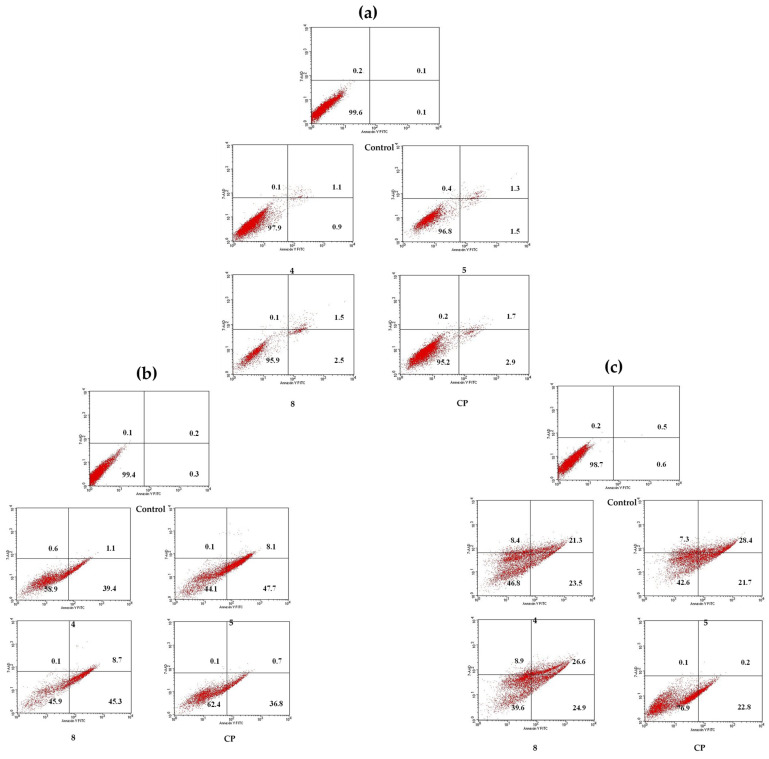
Representative results (%) as dot plots from apoptosis analysis of (**a**) RWPE-1, (**b**) LnCaP and (**c**) DU-145 cells after treatment with conjugates 4, 5 and 8 and free ciprofloxacin (CP) tested with flow cytometry. The lower right quadrant contains early apoptotic cells. The upper right quadrant represents late stage apoptotic cells.

For all three tested compounds used with concentration IC50 the number of apoptotic cancer cells was higher than of RWPE-1 normal cells (Table 4). In normal epithelial prostate cells conjugate (**8**) induced early apoptosis in the highest number of cells (2.6%) and compound (**4**) in the lowest number of cells (0.8%). Interestingly, the highest percentage of cells in early apoptosis was observed in the presence of unmodified ciprofloxacin (2.9%). The percentage of RWPE-1 cells that are in the late apoptotic phase or become necrotic by the tested compounds did not exceed 1.5% (Figure 4a).

The percentage of LNCaP cells in the early stage of apoptosis is many times greater than the number of cells in the late stage of apoptosis or necrosis. Compound (**5**) induced the highest percentage of early apoptotic cells—47.7%—and late apoptotic cells—8.8%. Similar percentages of early and late apoptotic cells exhibited compound (**8**) (45.3% and 7.9%, respectively), whereas compound (**4**) showed the lowest early apoptosis (38.4%) and very low level of late apoptosis 1.9%. Unconjugated ciprofloxacin induced less early apoptosis (26.8%) and did not affect the late apoptosis (Figure 4b).

In contrast to the LNCaP cell line, a higher percentage of the DU-145 cells were in the late apoptosis phase. The percentage of early apoptotic DU-145 cells was 23.5%, 23.9% and 24.1% for compounds **4**, **5** and **8**, respectively, while the percentage of cells in late apoptosis for these compounds were 24.7%, 30.5% and 29.8%. The induction of late apoptosis with these compounds was many times higher than in the presence of unmodified ciprofloxacin, where it was 0.3%. On the other hand, the percentage of cells in the early phase of apoptosis after CP treatment was similar to conjugated forms and accounted for 20.8% (Figure 4c).

#### 3.2.2. IL-6 Release

To determine the concentration of IL-6 in normal and cancer cells after treatment with the tested ciprofloxacin derivatives, the enzyme-linked immunoassay was performed.

The RWPE-1 line secreted very low IL-6 (1.0 pg/mL) as compared to the LNCaP and DU145 controls, where it accounted for 40.1 and 134.475 pg/mL, respectively. More than a two-fold decrease in IL-6 concentration (64.27 pg/mL) was observed in DU145 cells after conjugate (**5**) treatment. Conjugates **4** and **8** were not so effective and reduced the value of cytokine concentration only 1.5 and 1.2 times (86.72 and 109.9 pg/mL, respectively); however, the weakest effect exhibited unmodified ciprofloxacin, which only slightly decreased the IL-6 release to the value of 119.72 pg/mL.

**Figure 5:**
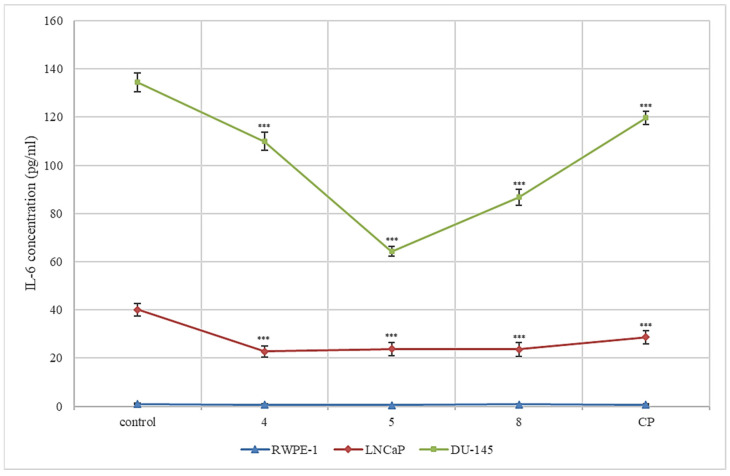
IL-6 concentration determined by ELISA method in the tested cell lines LNCaP, DU145 and RWPE-1 treated with new ciprofloxacin derivatives **4**, **5** and **8** and free ciprofloxacin (CP). Significance levels explanation *** *p* < 0.001 as compared to the controls.

The LNCaP cells showed a weaker secretion of IL-6 (40.1 pg/mL); however, conjugates **4**, **5** and **8** reduced the level of this interleukin almost twice, and the concentration values of IL-6 for individual compounds were 22.7, 23.6 and 23.8 pg/mL, respectively. No significant differences were observed between the effect of the conjugates and unmodified ciprofloxacin on the inhibition of the IL-6 secretion by LNCaP cells (28.7 pg/mL) (Figure 5).

## 4. A Summary of the Cancer Cell Lines Sensitivity to Individual Conjugates

Taking into account also the previous study on the PC3 cell line [24], we compared the activity of individual conjugates **2**, **4**, **5** and **8** on the tested cancer cell lines (LnCaP, DU-145 and PC3). The summary includes tests for the cytotoxic potential assessment and the cytotoxicity mechanisms on the basis of which we estimated the conjugates activity (Table 5).

We established a scale for estimating the efficiency of the tested conjugates in the form of the number of pluses, and so we marked the best efficiency with three pluses (+ + +, difference above 70% of control), the moderate with two pluses (+ +, difference from 30 to 70% of control) and the weakest with one plus (+, difference below 30% of control).

## 5. Proteomic Analysis

A comparative analysis of the effectiveness of selected conjugates showed that their cytotoxic activity was most pronounced in the PC3 cell line. Therefore, the proteomic assay was performed on the cancer PC3 and normal RWPE-1 cell lines. The conjugates **4, 5** and **8** with the highest cytotoxic potential were used for this analysis. Based on our previous study, conjugate (**2**) with sorbic acid was chosen as a good candidate for this assay (Table 6 and Table S1).

An almost four-fold down-regulation of lipid transporter fatty acid-binding protein 5 (FABP5) expression was observed for conjugate (**2**), whereas for the NPC intracellular cholesterol transporter 2 (NPC2) an about two-fold protein expression up-regulation was noticed. The conjugates **4** and **5** were weaker transport protein FABP5 reductors with a down-regulation of FABP5 protein expression by 40% and 12%, respectively. In contrast, the expression of the other transporter—NPC2—was down-regulated after treatment with both conjugates **4** and **5** by 10% and 30%**.** An evident up-regulation (from 1.5 to 3 times) in phospholipid-transporting ATPase IA (AT8A1) protein expression was noted after action of all conjugates. Reduced caveolin-1 (CAV1) expression was observed under action of conjugates **2**, **4** and **5** with the strongest activity noted for conjugate **5** (down-regulation by 40%). Only conjugate (**8**) slightly increased the expression of this protein (by 10%).

Generally, the key enzymes for fatty acid synthesis such as acetyl-CoA carboxylase 2 (ACACB) and fatty acid synthase (FASN) were depleted after the addition of conjugates, excluding the sorbic acid conjugate, where no radical changes were noticed. Conjugates **4** and **5** were the strongest reducing agents, due to a drop of nearly 80% of the carboxylase enzyme protein expression and of 60% and 80% for synthase enzyme protein, respectively. Conjugate (**8**) turned out to be a weaker reducer, as under its influence the expression of both enzymes was down-regulated by almost 40%. The weakest factor was conjugate (**2**), which only influenced the carboxylase production, reducing it by 20%.

The strongest perilipin-3 expression reductor (by 60%) was conjugate (**5**), whereas conjugates **2** and **4** reduced this lipid storage protein by about 20%. The conjugate (**8**) was the least pronounced in its influence on this protein (reduction by 9%).

Analysis of the mitochondrial catabolic lipid enzymes showed a decrease in both 2,4-dienoyl-CoA reductase (DECR-1) and acetyl-CoA acetyltransferase (ACAT1) expression after treatment with studied conjugates. The most pronounced DECR-1 and ACAT1 reductor was conjugate (**2**), which silenced expression of reductase and transferase by 65% and 50%, respectively, whereas conjugates **4** and **5** down-regulated expression of acetyl-CoA acetyltransferase by 50% and 35% and 2,4-dienoyl-CoA reductase by about 35% and 50%. Conjugate (**8**) reduced the expression of acetyltransferase much less (by 15%) and in the case of transferase, it even induced its expression by about 20%.

Changes in the expression of selected protein were not so evident in healthy RWPE-1 cells. The FABP5 transporter was not found in normal epithelial prostate cells, whereas the AT8A1 and CAV1 expression was slightly down-regulated. Similarly to cancer cells, an increase in the expression of AT8A1 was observed but not as pronounced. The expression of the anabolic enzyme FASN and the storage protein PLIN3 was up-regulated, as opposed to carboxylase ACACB which was down-regulated. Catabolic enzyme 2,4-dienoyl-CoA reductase expression was decreased after treatment with conjugates **2** and **4** and gently up-regulated after addition conjugates **5** and **8**. A noticeable increase in ACAT1 of expression after treatment with all tested conjugates was observed. The effect of ciprofloxacin alone was much weaker in both cancer and normal cell lines.

## 6. Discussion

Currently, numerous studies are conducted aimed at introduction of new compounds, ones which could replace or support the drugs usually used, into oncological therapy. Due to their potency, bioavailability and safe application profile, fluoroquinolones are used in the treatment of many types of bacterial infections. They have also been studied for their possible use as anti-cancer drugs. This fluoroquinolones application was based on their affinity for eukaryotic DNA topoisomerases [5,29]. In this respect, fluoroquinolones are considered as a safer, less cardiotoxic, alternative to conventionally used drugs such as etoposide and doxorubicin [30,31]. Ciprofloxacin, as a known fluoroquinolone, and depending on the time of administration and dose, may inhibit the growth or induces apoptosis of various cancer cell lines, including osteosarcoma and leukemia. In the last twenty years, more and more in vitro studies have showed the effect of ciprofloxacin on various tumor cell lines [6,32]. The effect of ciprofloxacin used in high concentrations (about 200 µg/mL) on the bladder and prostate cancer cell lines has been demonstrated in in vitro studies [6,33]. In addition, a comparison of the cytotoxic action of ciprofloxacin and levofloxacin on prostate and bladder cancer indicated a greater anticancer potential of ciprofloxacin [34].

Lipid metabolism and signaling alteration is characteristic for the prostate cancer malignant phenotype and includes the upregulation of several lipogenic and lipolytic enzymes as well as changes in cholesterol and phospholipid metabolism. It manifests in an increased lipid requirement, including the uptake of circulating free fatty acids by prostate cancer cells, as well as a raised de novo synthesis of fatty acids [35,36,37,38].

Considering the above, we have hypothesized that conjugation with fatty acid being “the Trojan horse” will potentiate the anti-cancer effect of ciprofloxacin. In our study, the potential antitumor properties of new ciprofloxacin conjugates with selected fatty acids were assessed. Among the tested compounds, three of them conjugated with oleic, elaidic and docosahexaenoic acids exhibited the highest anticancer effect.

To the best of our knowledge, as yet there are no studies about the influence of ciprofloxacin-fatty acids conjugates on cancer cell lines except our paper on the influence of ciprofloxacin on PC3 cell line [24]. Moreover, there are no research on the effect of fatty acids conjugates with other cytotoxic agents on prostate cancer cells. At the same time, we are aware that results of in vitro tests may not be fully translated into activity in the tumor, resulting in a significant obstacle to rational evaluation of the conjugates activity on cancer cells in vivo.

Another limitation of our study is the lack of knowledge on therapeutic effectiveness of the tested conjugates following oral administration considering that their decomposition by enzymes in the liver and gastrointestinal tract should be expected.

Thus, our results may primarily translate to the expected therapeutic effect of the conjugates after their parenteral administration.

However, drugs with an amide bond can also be completely resistant to in vivo hydrolysis and their biotransformation then takes place using oxidative pathways [39].

Oleic acid (OA) is a monounsaturated fatty acid which millimolar levels in the human plasma are steady and do not change with supplementation. Therefore, we do not discuss studies in vitro regarding the effect of OA alone on cancer cells. The most controversial results are from studies conducted on cancer cells with the oleic acid isomer-elaidic acid. The effect of modification of 5-azacytidine with elaidic acid (EA) was a much easier penetration into cancer cells. It allowed to omit the action of cytidine kinase on this chemotherapeutic agent and to obtain a better anticancer effect [40]. On the other hand, Ohmori et al. demonstrated that EA can increase the metastatic potential of colorectal cancer cells (CRC) to the liver, lungs and peritoneum. Moreover, they found that EA increased the resistance of these cells to 5-fluorouracil [41]. Pro-metastatic effects of EA in CRC have been associated with activation of EGFR signaling in cholesterol-rich lipid rafts [42], While, it was showed that the use of DHA with taxane drugs suppressed genes related to NF-κB. The results of this study suggest that the use of DHA with a drug from the taxane group weakens the resistance of cancer cells to taxanes and increases their cytotoxic effect on prostate cancer cells [43]. Another study proved the effect of DHA acid used in concentrations of 25 and 50 μg/mL on prostate cancer cells (PC3) by showing an inhibition of invasiveness and tumor cells migration, while higher concentrations (100 and 200 μg/mL) were shown to affect the cell proliferation [44]. Generally, the ability of DHA to induce apoptosis of various cancer cells or reduce their proliferation in vitro and in vivo is caused by changing the structure of biological membranes, lipid peroxidation, eicosanoid metabolites or by acting on nuclear receptors [45,46]. In turn sorbic acid is known as a food and cosmetic preservative, and some studies confirmed its lack of pro-cancerogenic properties [47,48]. However, its cytotoxic effect on cancer cells remains unknown.

Using the MTT test, we showed that the IC50 values for the ciprofloxacin-OA, EA and DHA conjugates were significantly lower for both cancer cell lines (LnCaP and DU145) than by using ciprofloxacin alone. Generally, all newly synthetized conjugates exhibited higher cytotoxicity than the free ciprofloxacin. This finding proved a greater anticancer potential of the conjugated form. Likewise, the use of separate conjugate components confirmed the better efficiency of the conjugated form. Based on our previous study, we observed that PC3 metastatic prostatic cells were also very strongly affected by those conjugates and their IC50 concentration was very low while the release of LDH increased several times compared to the control [24].

The conducted studies also showed that the selected ciprofloxacin-OA, EA and DHA conjugates induced early apoptosis in the LNCaP or early and late apoptosis in DU145 cell line. Remarkably, early and late apoptosis were induced by conjugates in hormone independent cancer cells DU145. In comparison, as we have established in our previous study, also the hormone-independent PC3 cells line was strongly driven into a late apoptosis state. Some neoplastic tissues are characterized by an increased IL-6 secretion, which is an indicator of aggressive cell growth and decrease cellular response to the applied therapy. High levels of IL-6 in the blood of cancer patients are associated with worse prognosis and survival [28]. The analysis of IL-6 concentrations showed that the ciprofloxacin-elaidic conjugate reduced the amount of secreted interleukin in DU145 cells the strongest. Significant differences between the activity of the conjugates and unmodified ciprofloxacin on the inhibition of interleukin 6 secretion were observed in DU-145 cells. The obtained results from LNCaP cells indicated that the individual conjugates OA, EA and DHA conjugates showed a similar suppressive effect on cytokine secretion. There was no spectacular difference in the effect between the conjugates and the unmodified ciprofloxacin in this type of cell line Secretion of interleukin in normal cells was very low and the effect of conjugates on interleukin concentration was very poorly evident.

Intracellular lipid chaperones that bind and facilitate the transport of long-chain fatty acids and related lipids to various cellular compartments are the fatty acid binding proteins (FABPs). Humans express ten distinct FABP isoforms and one of them termed FABP5 is not expressed in the normal prostate cells whereas it is highly upregulated in advanced metastatic PCa. FABP5 is a key transport protein, delivering cytosolic lipids to nuclear receptors to promote a metastatic PCa phenotype [49]. Using proteomic analyses, we showed that studied the conjugates, especially the combination with sorbic and oleic acid, significantly decreased FABP5 expression. The combination with elaidic acid turned out to be a weaker reducer, while the conjugation with DHA did not affect this protein. Interestingly, it seems that because of the amphiphilic nature of the conjugates, flippases may be involved in the transport of ciprofloxacin conjugates. These proteins normally transport phospholipids, but they also transport other amphiphilic drugs which form micelle-like particles in aqueous solution [50]. Phospholipid-transporting ATPase IA expression after treatment with elaidic, oleic and DHA conjugates was significant increased, whereas the influence of sorbic acid amide on this transport protein was not so pronounced. It is widely accepted that cancer cells need an excess of cholesterol as well as intermediates from cholesterol biosynthesis to maintain cell proliferation. The analysis of prostate cancer animal models revealed that plasma cholesterol elevation causes an accumulation of cholesterol in lipid rafts and consequently leads to reduced apoptosis and increased tumor growth via Akt signaling [51]. NPC intracellular cholesterol transporter 2 is observed in a few neoplasms, including prostate cancer. It is interesting that, in our study, the conjugate with elaidic acid lowered this transporter expression 1.5 times, while conjugation with oleic acid lowered it only slightly (1.1 times). On the contrary, conjugates with both sorbic and DHA acids increased the expression of this transport protein, and in the case of sorbic acid it was almost twice. An integral membrane protein of lipid rafts important for signal transduction is caveolin-1 (CAV1). Depending on the stage and type of tumor, the role of CAV-1 in cancer is ambiguous. It can act as a tumor suppressor or a metastasis promoter; however, in prostate cancer it can stimulate cell survival and angiogenic activities [52,53].

Among lipogenic enzymes, the fatty acid synthase (FASN) has been identified as oncogene product. The direct FASN product is palmitate—the saturated fatty acid. The FASN overexpression is consistently found in prostate tumors. It turned out that in prostate cancer the FA proportions change with increased amount of monounsaturated fatty acids [54]. The tested conjugates sorbic acid conjugate, significantly reduced expression of FAS. The strongest reducer turned out to be the conjugate with elaidic acid, decreasing the FAS expression by as much as 80%, also OA and DHA conjugates significantly reduced the intensity of this enzyme protein by 50% and 40%, respectively.

A very similar scenario of the conjugates activity was noticed with a different regulatory lipogenic enzyme Acetyl-CoA carboxylases (ACC). Two ACC isoforms have been identified in mammals, i.e., ACC-alpha (ACCA, also termed ACC1) and ACC-beta (ACCB, also designated ACC2). ACC up-regulation for increased lipid requirements has been showed in various cancers [55]. Both conjugates **4** and **5** strongly reduced ACCB level by 80%. A weaker reducer was the DHA conjugate (by 45%) and the weakest conjugate was sorbic acid (by 18%).

In terms of lipid storage, perilipin is a known protein involved in this process. Abnormal lipid droplet (LDs) storage is a common phenomenon in neoplastic cells. LDs consist of a neutral lipid core with triglycerides and cholesterol esters, while the outer monolayer is comprised of phospholipids and surface proteins such as perilipin-3 (PLIN3). High PLIN3 expression in prostate cancer was positively correlated with tumor stage and Gleason score. Lipid accumulation in prostate cancer cells accelerates androgen synthesis, which can promote AR reactivation and/or abnormal activation, leading to CRPC phenotype [56,57]. Our tested conjugates successfully lowered the level of the PLIN3 protein, and among them EA conjugate was the most active (43% of control), while after conjugate treatment with sorbic and oleic acid PLIN3 intensity accounted approximately for 80% of control.

Similarly to anabolic enzymes, the enzyme proteins associated with lipid catabolism decreased in expression after treatment with conjugates. The level of mitochondrial acetyl-CoA acetyltransferase (ACAT1) was halved when the cells were treated with sorbic and elaidic conjugates, whereas after adding OA conjugate it was 70% of control. Only after treatment with the DHA conjugate the expression of ACAT1 slightly increased. It was found that ACAT1 may regulate enzymatic complex by acetylating pyruvate dehydrogenase (PDH) and PDH phosphatase. Decreased ACAT1 activity leads to impaired cancer cell proliferation and tumor growth [58]. The level of another catabolic enzyme 2,4-dienoyl-CoA reductase (DECR1) was reduced by 70% in the presence of sorbic conjugate and by almost 50% under treatment with OA and EA conjugates. The DHA conjugate was a weaker reducer with a decrease in protein level of 35%. DECR1 is a mitochondrial enzyme engaged in the accessory pathway of beta-oxidation as well as it is a key regulator of polyunsaturated fatty acids entering the catabolic pathway within the mitochondria. The DECR1 function is controversial in cancer cells. Its overexpression can protect cancer cells from glucose deprivation-induced apoptosis. In the other hand, DECR1 was recognized as a cancer suppressor in HER2-positive breast cancer [59]. Moreover, DECR1 controls the ratio between saturated and unsaturated phospholipids and thus redox homeostasis. DECR1 deletion activates endoplasmic reticulum (ER) stress and sensitizes CRPC cells to ferroptosis. In vivo, DECR1 knockout attenuates lipid metabolism and reduces CRPC tumor growth [60].

## 7. Conclusions

All nine ciprofloxacin fatty acid conjugates analyzed in the preliminary evaluation showed high cytotoxicity against cell line (DU145) and hormone-sensitive (LNCaP) prostate cancer but not for normal prostate cell line (RWPE1). Among the tested fatty acids related to ciprofloxacin, the most powerful ones are oleic, elaidic and docosahexaenoic acids. All above are unsaturated long chain acids. Based on our previous research, we noticed that conjugate **2** is a promising compound, although unlike the previously mentioned acids, this one belongs to the family of short-chain acids and has two double bonds. The hormone-non-sensitive prostate cancer cell line DU145 appears to be more susceptible to the new synthesized ciprofloxacin derivatives than the hormone-sensitive LNCaP line. Similarly, our previous study confirmed a stronger cytotoxic effect of selected conjugates on more invasive and non-sensitive hormone cell line PC3. The newly synthesized conjugates appear to be more effective in more invasive prostate cancer cells that are resistant to hormone therapy. Interestingly, the proteins involved in lipid metabolism, which have been identified as key molecules in the prostate carcinogenesis and the achievement of an androgen-insensitive phenotype, have been silenced by our tested conjugates. It seems to confirm the beneficial anti-cancer effect of the tested ciprofloxacin conjugates in prostate cancer. Summarizing the obtained results, the most optimal combination seems to be a conjugation of ciprofloxacin with oleic acid and elaidic acid. Taken together, these findings validate the importance of the lipid metabolism in prostate carcinogenesis and identify a target for potential novel therapeutic strategies and drug carriers.

## Figures and Tables

**Table 1:** Fatty acids being substituents in the structure of the tested compounds.

N^o^	Substituent (R)	The Substituent Name (Common/IUPAC)	Chain Length/Number of Unsaturation/Isomer
1	C_3_H_5_C(O)-	Crotonic acid (2E)-But-2-enoic acid	4:1 (E2)
2	C_5_H_7_C(O)-	Sorbic acid (2E,4E)-hexa-2,4-dienoic acid	6:2 (E2, E4)
3	C_9_H_15_C(O)-	Geranic acid (2E)-3,7-Dimethyl-2,6-octadienoic acid	10:2 (E2, E6)
4	C_17_H_33_C(O)-	Oleic acid (9Z)-Octadec-9-enoic acid	18:1 (Z9)
5	C_17_H_33_C(O)-	Elaidic acid (E)-octadec-9-enoic acid	18:1 (E9)
6	C_17_H_29_C(O)-	Linolenic acid (9Z,12Z,15Z)-octadeca-9,12,15-trienoic acid	18:3 (Z9, Z12, Z15)
7	C_21_H_41_C(O)-	Erucic acid (Z)-Docos-13-enoic acid	22:1 (Z13)
8	C_21_H_31_C(O)-	Docosahexaenoic acid (4Z,7Z,10Z,13Z,16Z,19Z)-docosa-4,7,10,13,16,19-hexaenoic acid	22:6 (Z4, Z7, Z10, Z13, Z16, Z19)
9	C_15_H_31_C(O)-	Palmitic acid Hexadecanoic acid	16:0

**Table 2:** The IC50 and SI values of the new ciprofloxacin conjugates in cancer and normal prostate cells.

Compound	Cancer Cells						Normal Cells
	LNCaP ^c^		DU-145 ^d^		PC3 ^e^		RWPE-1 ^f^
	IC_50_^a^	SI ^b^	IC_50_	SI	IC_50_	SI	IC_50_
**1**	66.7 ± 7.9	1.4	62.4 ± 5.6	1.4	93.8 ± 3.4	0.9	81.2 ± 6.9
**2**	38.8 ± 3.2	1.6	24.7 ± 5.3	2.5	11.7 ± 1.8	5.3	62.5 ± 6.4
**3**	39.5 ± 3.1	1.9	33.8 ± 2.1	2.3	73.4 ± 2.7	1.1	78.1 ± 3.6
**4**	24.7 ± 4.1	2.7	20.2 ± 1.9	3.3	7.7 ± 2.1	8.7	66.9 ± 3.8
**5**	22.4 ± 3.1	2.9	17.8 ± 1.6	3.6	15.3 ± 5.3	4.2	64.8 ± 5.9
**6**	39.8 ± 4.6	1.4	25.9 ± 2.4	1.8	34.4 ± 2.4	1.7	57.2 ± 6.7
**7**	59.2 ± 4.3	1.0	27.6 ± 2.4	2.2	76.3 ± 3.3	0.8	60.8 ± 5.3
**8**	21.4 ± 2.2	3.0	16.5 ± 1.4	4	27.7 ± 1.9	2.3	65.1 ± 2.2
**9**	39.0 ± 4.7	1.3	48.1 ± 2.3	1.1	51.08 ± 4.5	1.0	51.3 ± 4.6
Ciprofloxacin ^g^	71.2 ± 3.8	1.0	70.5 ± 3.6	1.0	101.38 ± 3.6	0.7	72.1 ± 5.2
Cisplatin ^h^	1.78 ± 0.74	1.5	1.36 ± 0.69	2	1.51 ± 0.24	1.78	2.69 ± 1.1
Doxorubicin ^h^	0.48 ± 0.21	1.3	0.59 ± 0.14	1.1	0.31 ± 0.12	2	0.65 ± 0.1

^a^ The IC_50_ value is defined as the concentration of a compound that corresponds to a 50% growth inhibition. Data are expressed as mean ± SD. ^b^ The SI (selectivity index) was calculated for each compounds using the formula: SI = IC_50_ for normal cell line/IC_50_ cancer cell line. ^c^ LNCaP—human metastatic prostate cancer cells (derived from left supraclavicular lymph node), ^d^ DU145—human metastatic prostate cancer cells (derived from brain), ^e^ PC3—human metastatic prostate cancer cells (derived from bones), ^f^ RWPE-1—normal human epithelial prostate cells, ^g^ CP—parent ciprofloxacin; ^h^ referenced cytostatic.

**Table 3:** The IC50 and SI values of the tested unconjugated ciprofloxacin and individual fatty acids.

Compounds	Cancer Cells						Normal Cells
	LNCaP ^c^		DU145 ^d^		PC3 ^e^		RWPE-1 ^f^
	IC_50_ ^a^	SI ^b^	IC_50_	SI	IC_50_	SI	IC_50_
CA ^g^ + CP ^h^	65.6 ± 6.9	1.1	75.2 ± 4.8	0.9	79.2 ± 3.4	0.9	73.1 ± 5.7
SA + CP	69.1 ± 7.4	1.0	69.3 ± 7.3	1.0	68.4 ± 2.8	1.1	73.2 ± 9.5
GA + CP	69.3 ± 8.5	0.9	76.1 ± 8.3	0.9	72.2 ± 5.8	0.9	66.3 ± 9.2
OA + CP	51.8 ± 4.9	1.2	50.7 ± 4.8	0.9	67.3 ± 4.7	0.9	60.2 ± 9.3
EA + CP	37.7 ± 5.5	1.2	31.4 ± 5.2	1.4	66.3 ± 5.1	0.7	46.3 ± 5.9
LA + CP	61.7 ± 7.3	0.9	51.6 ± 4.9	1.1	58.2 ± 3.5	1.0	55.9 ± 6.7
EA + CP	54.2 ± 6.5	1.1	58.6 ± 6.5	1.0	79.3 ± 6.2	0.8	61.6 ± 8.4
DHA + CP	38.4 ± 4.7	1.3	36.9 ± 3.7	1.3	43.5 ± 3.7	1.1	49.7 ± 6.3
PA + CP	37.7 ± 9.7	1.3	42.5 ± 6.4	1.1	61.23 ± 4.2	0.8	49.3 ± 5.8
CP	71.2 ± 3.8	1.0	70.5 ± 3.6	1.0	101.38 ± 3.6	0.7	72.1 ± 5.2

^a^ The IC_50_ value is defined as the concentration of compounds that corresponds to a 50% growth inhibition. Data are expressed as mean ± SD. ^b^ The SI (selectivity index) was calculated for each compounds using formula: SI = IC_50_ for normal cell line/IC_50_ cancer cell line. ^c^ LNCaP—human metastatic prostate cancer cells (derived from left supraclavicular lymph node), ^d^ DU145—human metastatic prostate cancer cells (derived from brain), ^e^ PC3—human metastatic prostate cancer cells (derived from bones), ^f^ RWPE-1—normal human epithelial prostate cells, ^g^ free fatty acid—CA—crotonic acid; SA—sorbic acid; GA—geranic acid; OA— acid; EA—elaidic acid; LA—linolenic acid; EA—erucic acid; DHA—docosahexaenoic acid; PA—palmitic acid; ^h^ CP—unmodified ciprofloxacin.

**Table 4:** The effect of new ciprofloxacin derivatives **4**, **5**, **8** and * free ciprofloxacin on early or late apoptosis in studied cancer (LNCaP and DU145) and normal RWPE-1 cell lines detected with Annexin V-FITC/7-ADD by flow cytometry ^a^ % of cells in early apoptotic state; ^b^ % of cells in late apoptotic state.

Cell Line	Compound	Early Apoptosis ^a^ (%)	SD	Late apoptosis ^b^ (%)	SD
**Control**	-	0.2	0.1	0.2	0.1
RWPE-1	4	0.8	0.3	1.3	0.7
	5	1.3	0.9	1.5	0.5
	8	2.6	1.1	1.5	0.9
	CP *	2.9	1.1	1.4	0.4
**Control**	-	0.3	0.1	0.2	0.1
LNCaP	4	38.4	3.3	1.9	0.8
	5	47.7	4.7	8.8	1.8
	8	45.3	3.9	7.9	2.2
	CP	26.8	3.9	0.7	1.1
**Control**	-	0.6	0.1	0.5	0.2
DU145	4	23.5	3.5	24.7	3.5
	5	23.9	2.6	30.5	3.6
	8	24.1	2.1	29.8	4.9
	CP	20.8	3.5	0.3	0.2

**Table 5:** The comparison of prostate cancer cell lines LnCaP, DU145 and PC3 sensitivity for selective conjugates **2**, **4**, **5** and **8** based on the results from cytotoxicity tests (MTT and LDH), apoptosis assay and IL-6 release inhibition.

	Compound	LnCaP	DU145	PC3
**MTT test**	**2**	**+**	**+ +**	**+ + +**
	**4**	**+ +**	**+ +**	**+ + +**
	**5**	**+ +**	**+ + +**	**+ + +**
	**8**	**+ +**	**+ + +**	**+ +**
**LDH test**	**2**	**+**	**+**	**+ + +**
	**4**	**+ +**	**+ +**	**+ + +**
	**5**	**+ + +**	**+ + +**	**+ + +**
	**8**	**+ + +**	**+ + +**	**+ + +**
**Apoptosis assay**	**2**	**+**	**+**	**+ + +**
	**4**	**+ +**	**+ +**	**+ + +**
	**5**	**+ +**	**+ +**	**+ + +**
	**8**	**+ +**	**+ ++**	**+ + +**
**IL-6 release inhibition**	**2**	**+**	**+**	**+ + +**
	**4**	**+ +**	**+ +**	**+ + +**
	**5**	**+ +**	**+ + +**	**+ + +**
	**8**	**+ +**	**+ + +**	**+**

+, difference below 30% of control. + +, difference from 30 to 70% of control. + + +, difference above 70% of control

**Table 6:** LC-MS proteome analysis in PC3 and RWPE-1 cells treated with IC_50_ concentrations of conjugates **2, 4, 5** and **8** and free ciprofloxacin. Protein expression was normalized to the control (given as 100%) and shown as a percentage of a control.

Accession	Name of Enzyme	PC3						RWPE-1					
		Control	2	4	5	8	CP	Control	2	4	5	8	CP
Lipid transport and signaling													
FABP5_ HUMAN	Fatty acid-binding protein 5	100	26.2	60.8	88.9	100.8	92.4	-	-	-	-	-	-
AT8A1_ HUMAN	Phospholipid-transporting ATPase IA	100	142.2	247	313.9	249.5	124.5	100	111.2	109.3	114.5	107.6	121.2
NPC2_ HUMAN	NPC intracellular cholesterol transporter 2	100	184.2	93.3	69.5	131.7	91.3	100	92.7	98.2	94.7	94.5	91.2
CAV1_ HUMAN	Caveolin-1	100	94.3	86.2	59.1	113.8	89.2	100	94.8	87.1	85.3	82.7	92.5
Lipid anabolism and storage													
FAS_HUMAN	Fatty acid synthase	100	101.1	48.1	24.1	62.4	87.4	100	122.1	113.6	118.6	106.6	94.5
ACACB_ HUMAN	Acetyl-CoA carboxylase 2	100	88.2	21.4	22.8	56.5	89.3	100	89.4	96.4	93.0	83.9	89.3
PLIN3_ HUMAN	Perilipin-3	100	75.2	81.1	42.7	91.2	86.3	100	105.2	113.4	116.2	132.3	95.2
Lipid catabolism													
DECR_ HUMAN	2,4-dienoyl-CoA reductase, mitochondrial	100	35.5	51.7	64.4	84.7	82.7	100	91.3	92.5	109.2	102.4	96.3
THIL_ HUMAN	Acetyl-CoA acetyltransferase, mitochondrial	100	54.2	71.2	51.6	119.1	83.4	100	123.4	132.3	129.3	108.3	94.6

## Data Availability

https://biochemia.wum.edu.pl/ (accessed on 30 September 2021); https://medycynaregeneracyjna.wum.edu.pl/ (accessed on 30 September 2021); Raw data is available under request. In order to get access to raw data please contact to corresponding author.

## Supplementary Materials

The following supporting information can be downloaded at: https://www.mdpi.com/article/10.3390/cancers14020409/s1, Table S1: LC-MS proteome analysis provided in the cancer PC3 and normal RWPE-1 cells treated for 72 h with IC_50_ concentrations of CP amides **2, 4, 5** and **8** and free CP. Protein intensities were expressed as a mean from three independent experiments.

## Abbreviations

PCa	prostate cancer
CRPC	castration-resistant PCa
CP	Ciprofloxacin
FA	fatty acid
LDs	lipid droplets
AR	androgen receptor
IL-6	interleukin 6
OA	oleic acid
EA	elaidic acid
DHA	docosahexaenoic acid
NF-κB	nuclear factor-κB
FITC	fluorescin
EGFR	epidermal growth factor receptor
