# Supplementary material for: The Effect of Fatty Acids on Ciprofloxacin Cytotoxic Activity in Prostate Cancer Cell Lines—Does Lipid Component Enhance Anticancer Ciprofloxacin Potential?
Source: Cancers (Basel). 2022 Jan 14;14(2):409. doi: 10.3390/cancers14020409 (PMC8773529; doi:10.3390/cancers14020409)
Supplement: Supplementary file 1 [file cancers-14-00409-s001.zip › cancers-1503709-supplementary.pdf]

cancers

MDPI

Supplementary material:

Table S1. LC-MS proteome analysis provided in the cancer PC3 and normal RWPE-1 cells treated for 72h with IC_{50} concentrations of CP amides **2, 4, 5** and **8** and free CP. Protein intensities were expressed as a mean from three independent experiments.

Accession	Name of enzyme	PC3						RWPE-1					
		Control	2	4	5	8	CP	Control	2	4	5	8	CP
		**Lipid transport and signaling**											
FABP5_HUMAN	**Fatty acid-binding protein** 5 OS=Homo sapiens OX=9606 GN=FABP5 PE=1 SV=3	2099906	550175.4	1276742.1	1866816.4	2133639	1940313	-	-	-	-	-	-
AT8A1_HUMAN	**Phospholipid-transporting ATPase** IA OS=Homo sapiens OX=9606 GN=ATP8A1 PE=1 SV=1	115840	164724.5	286124.8	363621.8	289020.8	144220.8	98750	109810	107933.7	113068.7	106 235	119685
NPC2_HUMAN	**NPC intracellular cholesterol transporter 2** OS=Homo sapiens OX=9606 GN=NPC2 PE=1 SV=1	90108	165978	84070.8	62625.1	118672.2	82268.6	60306	55903.6	59220.5	57109.8	56989.2	54999.1
CAV1_HUMAN	**Caveolin-1** OS=Homo sapiens OX=9606 GN=CAV1 PE=1 SV=4	725820	684448.3	625656.8	428959.6	825983.2	647431.4	64740	61373.5	56388.6	55223.2	53539	59884.5
		**Lipid anabolism and storage**											
FAS_HUMAN	**Fatty acid synthase** OS=Homo sapiens OX=9606 GN=FASN PE=1 SV=3	1125936	1138321.3	541575.2	271350.6	702584.1	984068.1	823421	1005397	935406.2	976577.3	877766.8	778132.9
ACACB_HUMAN	**Acetyl-CoA carboxylase 2** OS=Homo sapiens OX=9606 GN=ACACB PE=1 SV=3	253760	223816.3	54304.6	57857.3	143374.4	226607.7	49340	44109	47563.7	45866.2	41396.3	44060.6
PLIN3_HUMAN	**Perilipin-3** OS=Homo sapiens OX=9606 GN=PLIN3 PE=1 SV=3	960327	722165	778825.2	410059.6	875818.2	828762.2	631231	664055	715815	733490.4	835118.6	600932
		**Lipid catabolism**											
DECR_HUMAN	**2,4-dienoyl-CoA reductase,** mitochondrial OS=Homo sapiens OX=9606 GN=DECR1 PE=1 SV=1	192442	68317	99492.5	123932.6	162998.4	159149.5	111503	101802.2	103140.3	121761.3	114179.1	107377.4
THIL_HUMAN	**Acetyl-CoA acetyltransferase,** mitochondrial OS=Homo sapiens OX=9606 GN=ACAT1 PE=1 SV=1	79046	42843	56280.7	40787.7	94143.8	65924.4	69548	85822.2	92012	35886.8	82831.7	58003
